# Rab27a Contributes to the Processing of Inflammatory Pain in Mice

**DOI:** 10.3390/cells9061488

**Published:** 2020-06-18

**Authors:** Tilman Gross, Gesine Wack, Katharina M. J. Syhr, Tanya Tolmachova, Miguel C. Seabra, Gerd Geisslinger, Ellen Niederberger, Achim Schmidtko, Wiebke Kallenborn-Gerhardt

**Affiliations:** 1 Institute of Pharmacology and Clinical Pharmacy, Goethe University, 60438 Frankfurt am Main, Germany; t.gross@em.uni-frankfurt.de (T.G.); wack@em.uni-frankfurt.de (G.W.); schmidtko@em.uni-frankfurt.de (A.S.); 2 Pharmazentrum Frankfurt/ZAFES, Institute of Clinical Pharmacology, Goethe University, 60596 Frankfurt am Main, Germany; katharina.syhr@googlemail.com (K.M.J.S.); geisslinger@em.uni-frankfurt.de (G.G.); e.niederberger@em.uni-frankfurt.de (E.N.); 3 Molecular Medicine Section, National Heart and Lung Institute, Imperial College London, London SW7 2AZ, UK; tanya.tolmachova@gmail.com; 4 CEDOC, NOVA Medical School, Universidade NOVA de Lisboa, 1169-056 Lisbon, Portugal; miguel.seabra@nms.unl.pt; 5 Fraunhofer Institute for Molecular Biology and Applied Ecology (IME), Branch Translational Medicine and Pharmacology, 60595 Frankfurt am Main, Germany

**Keywords:** Rab27a, inflammatory pain, spinal cord, dorsal root ganglia, mice

## Abstract

Tissue injury and inflammation may result in chronic pain, a severe debilitating disease that is associated with great impairment of quality of life. An increasing body of evidence indicates that members of the Rab family of small GTPases contribute to pain processing; however, their specific functions remain poorly understood. Here, we found using immunofluorescence staining and in situ hybridization that the small GTPase Rab27a is highly expressed in sensory neurons and in the superficial dorsal horn of the spinal cord of mice. Rab27a mutant mice, which carry a single-nucleotide missense mutation of Rab27a leading to the expression of a nonfunctional protein, show reduced mechanical hyperalgesia and spontaneous pain behavior in inflammatory pain models, while their responses to acute noxious mechanical and thermal stimuli is not affected. Our study uncovers a previously unrecognized function of Rab27a in the processing of persistent inflammatory pain in mice.

## 1. Introduction

Chronic pain is a major source of suffering and one of the most frequent causes for patients to seek medical care [1]. Currently available treatments may be associated with sometimes severe side effects and are often only partially effective. Thus, it is important to elucidate molecular mechanisms of nociceptive processing in order to develop novel strategies for chronic pain treatment [2,3,4]. 

Accumulating evidence suggests that various Rab proteins contribute to pain signaling, but their specific functions remain poorly understood. In general, Rab proteins belong to the Ras superfamily of small guanosine triphosphatases (GTPases). As monomeric G-proteins they exist in a GTP and a GDP-bound state and their activity is regulated by guanosine nucleotide dissociation inhibitors and guanine nucleotide exchange factors (for review see [5]). More than 60 Rab proteins have been identified, which are involved in basic cellular processes such as cellular trafficking, vesicular trafficking and synaptic plasticity. In a previous study, we found that Rab7 contributes to the processing of inflammatory pain in a redox-dependent manner [6]. Moreover, expression of Rab7 is upregulated after peripheral nerve injury [7] and Rab7-mediated lysosomal targeting of µ-opioid receptors leads to a loss in opioid antinociception in diabetic neuropathic pain [8]. Furthermore, trafficking of the neurokinin 1 receptor (NK1R) in sarcoma virus-transformed rat kidney epithelial cells was found to be controlled by Rab5a [9] and expression of Rab23 was induced in an inflammatory pain model [10], suggesting that, also, other Rab proteins contribute to the processing of pain.

Another Rab protein that might be involved in pain signaling is Rab27a. Previous studies demonstrated that Rab27a regulates the motility and exocytosis of lysosome-related organelles and secretory granules in distinct cells; e.g., melanosomes in melanocytes, lytic granules in cytotoxic T cells and dense granules in platelets [11]. Mutations in the gene encoding for Rab27a result in the Griscelli Syndrome Type 2 (GS2), an autosomal recessive pigmentation disorder that is associated with severe immunological defects, caused by impaired function of neutrophil and natural killer cells as well as by malfunctioning cytotoxic T-lymphocytes [12,13,14,15]. In line with this, Rab27a mutant mice (Rab27a^ash/ash^) show dysfunction of melanosomes in melanocytes as well as impaired granule exocytosis of neutrophils [16,17,18,19]. 

Interestingly, some studies also suggest a neural expression of Rab27a. For example, Rab27a was found to be upregulated in cholinergic basal forebrain neurons in mild cognitive impairment and Alzheimer’s disease [20] and silencing Rab27a in primary hippocampal neuronal cultures resulted in attenuated calcium release from intracellular acidic calcium stores probably by attenuated exocytotic insertion of N-type calcium channels into the plasma membrane [21]. Furthermore, Rab27a has been shown to be expressed in the cortex and hippocampus where it probably controls exosome secretion [22]. Other studies provide evidence for a Rab27a expression in Schwann cells, in which it seems to regulate myelination processes of dorsal root ganglia in vivo [23,24], further supporting the idea that Rab27a exerts its functions not only in melanocytes and immune cells but also in the nervous system. However, despite its obvious neuronal expression, only little is known about the functional role of Rab27a in nociception. In this study we investigated the expression of Rab27a in nociceptive tissues and characterized its role in pain processing.

## 2. Materials and Methods

### 2.1. Animals

Generation of mice bearing a spontaneous single nucleotide mutation of Rab27a (Rab27a^ash/ash^), designated ashen [19,25], on a C57BL/6J background has been described previously [26]. Rab27a^ash/ash^ and wild type mice from heterozygous breeding were used. Genotyping was performed with gene specific primers (5′-ACCTGACAAATGAGCAAAGTTTCCTCAATG-3′ and 5′-GGAGCAGGGCAGGGCTGGGGAAACCACTCG-3′) followed by enzymatic restriction analysis with TaqI and RsaI [26]. In addition, tissue expression studies were performed in male C57BL/6N mice (Jackson Laboratories/Charles River, Sulzfeld, Germany). Animals were housed on a 12/12 h light/dark cycle with free access to water and food. All experiments were approved by the local Ethics Committee for Animal Research (Regierungspräsidium Darmstadt, Germany, approval number # V 54 – 19 c 20/15 – FU/1105 (2/2/2016)) and adhered to the guidelines of the Committee for Research and Ethical Issues of the International Association for the Study of Pain.

### 2.2. Behavior

Experiments were performed using littermate male and female mice at the age of 2 to 5 months. Rab27a mutant mice have a lightened, gray coat color because of defects in pigment granule transport, whereas wild type (WT) control mice have a black coat color. Therefore, various attempts were made to ensure the blinding of observers in behavioral experiments. Some experiments (formalin test, dynamic weight bearing) were video-taped and analyzed with increased contrast to prevent recognition of the coat color. All other experiments were performed by at least two observers in different experimental groups to confirm the tendency of the experimental outcome between observers.

Rotarod test. A rotarod treadmill for mice (Ugo Basile, Varese, Italy) was used to assess motor coordination, running at a constant rotating speed of 12 rpm. After several training sessions, fall-off latencies from 3 to 5 tests were averaged. The cut-off time was set at 120 s. 

Tape response assay. The tape response assay was performed to investigate basal touch sensitization in Rab27a mutant mice [27]. A common lab tape (3 cm long) was placed gently on the back of the mouse and the number of responses in 10 min were measured. A bite into the tape, shaking (wet-dog shake) or scratching the tape was considered as a positive reaction. 

Hot plate test. Mice were placed in an acrylic glass cylinder on a heated plate (50, 52, and 54 °C) and the latency time to the first nociceptive reaction (licking of the hind paws, shaking of the hind paws, jumping on the edge of the plexiglass cage) was measured [28]. Measurement at different temperatures was performed as the extent of activation of different heat receptors and the processing of thermal stimuli depend on the intensity of the stimulus [29,30]. In order to avoid tissue damage, cutoff times of 60 s, 40 s, and 20 s, respectively, were chosen. 

Dynamic plantar test. Mechanical paw withdrawal latencies were determined with a Dynamic Plantar-Aesthesiometer (Ugo Basile). The device consists of a stainless steel probe (0.5 mm diameter), which is pressed against the plantar surface of the paw (sural nerve skin area) by increasing force through a wire mesh bottom. A clear paw withdrawal was evaluated as a reaction. The force acting on the paw was increased from 0 to 5 g over a period of 10 s (0.5 g/s ramp) and then remained constant at 5 g for further 10 s (total switch-off time, 20 s). The latency was calculated as the average of four consecutive exposures with at least 20 s in between [31,32,33].

Formalin test. A 0.5% formaldehyde solution (15 µl; formalin) was injected subcutaneously into the dorsal site of one hind paw [34,35]. The time spent licking the formalin-injected paw was recorded in 5 min intervals up to 60 min after formalin injection.

Zymosan- and Complete Freund′s Adjuvant (CFA)-induced paw inflammation. Induction of paw inflammation by zymosan and CFA has been described previously [6,36]. Briefly, 15 µL of a zymosan A suspension (5 mg/mL in 0.1 M PBS, pH 7.4; Sigma-Aldrich, Steinheim, Germany) or 20 µL of complete Freund‘s adjuvant (CFA; containing 1 mg/mL heat-killed Mycobacterium tuberculosis in paraffin oil 85% and mannide monooleate 15%; Sigma-Aldrich) was injected into the plantar subcutaneous space of a hind paw [37]. To assess zymosan-induced mechanical hyperalgesia, the Dynamic plantar test was used as described above. CFA-induced inflammatory pain behavior was investigated using an automated dynamic weight bearing (DWB) device (Bioseb, Boulogne, France). The device consists of an acrylic glass cage (11 cm wide × 11 cm long × 20 cm high) and an instrumented floor to measure the weight borne in each limb. A camera is installed above the cage to validate the animal positions during the recording. During a habituation phase of 5 min and a recording phase of 5 min the mice were able to move freely in the cage. After a training session one day before the experiment, the DWB assessment was performed in naive mice (baseline) and after CFA injection, daily, for a period of 7 days. Following completion of each test, mice were removed and the test chamber was cleaned with water. The recorded weight distribution of the ipsilateral and contralateral hind paw was analyzed by using the DWB software as described [38]. A zone was considered as valid when the following parameters were detected: ≥0.8 g on one captor with minimum of two adjacent captors recording ≥1.0 g. A time segment was considered as valid if ≥3 stable pictures were detected. In all video sequences, the positions of the paws were manually validated by an observer. The mean of the weight over the time was used to calculate the ipsilateral hind paw to contralateral hind paw ratio.

### 2.3. Immunohistochemistry

Tissue preparation and immunofluorescence staining were performed as described previously [6,31,36]. Shortly, mice were intracardially perfused with 4% paraformaldehyde in PBS (pH 7.4) and the lumbar spinal cord (L4–L5) and dorsal root ganglia (DRGs) (L4–L6) were dissected, post-fixed, and cryoprotected in 20% sucrose overnight. Tissues were cryostat-sectioned at a thickness of 14–16 µm and stored at −80 °C. For immunofluorescence, sections were permeabilized in PBST (0.1% Triton-X in PBS), blocked in normal goat or donkey serum and 3% bovine serum albumin (BSA) in PBS, and incubated with primary antibodies diluted in 3% BSA in PBS over night at 4 °C or for 2 h at room temperature. The following antibodies were used: rabbit anti-Rab27a (1:200; Synaptic Systems, Goettingen, Germany # 168013; immunogen consists of a rat Rab27a fusion protein which shows 99% homology to murine Rab27a (National Center for Biotechnology Information (NCBI) # AAG24638.1) but only 71% homology to murine Rab27b (NCBI # AF02885.1)), mouse anti-calcitonin gene-related peptide (CGRP) (1:200; Sigma-Aldrich # C9487), mouse anti-neurofilament 200 (NF200) (1:2000; Sigma-Aldrich # N0142) and anti-NeuN (1:1000; Synapic Systems # 266004). Sections were then washed in PBS and stained with secondary antibodies conjugated with Alexa Fluor 488 (Invitrogen, Carlsbad, CA, USA), Cy3 (Sigma-Aldrich) or Cy5 (Invitrogen). For staining with Griffonia simplicifolia isolectin B4 (IB4), sections were incubated with Alexa Fluor 488-conjugated IB4 (10 µg/mL in PBS; Invitrogen) for 2 h at room temperature. After immunostaining, slides were immersed for 5 min in 0.06% Sudan black B (in 70% ethanol) to reduce lipofuscin-like autofluorescence [39,40], washed in PBS and coverslipped. Control experiments were performed in which the primary antibody was omitted. Images were taken using a Nikon Eclipse Ni-U microscope equipped with a monochrome DS-Qi2 camera (Nikon, Minato, Tokyo, Japan). Final adjustment of contrast and intensity were done using Adobe Photoshop software. Images taken from Alexa Fluor 488 stainings are presented in green, whereas images taken from Cy3 or Cy5 stainings are presented in magenta or yellow, respectively.

To analyze DRG neuron subpopulations, the percentage of neurons positive for marker (IB4, CGRP and NF200) was calculated from 3–4 animals per genotype (>1000 cells per genotype and marker). Only DRG neurons showing staining clearly above background were included. Sections were chosen randomly with at least 70 µm apart.

### 2.4. In Situ Hybridization

Mice were perfused with 4% formaldehyde (PFA) in PBS for 5 min. Lumbar (L4–L5) spinal cords and DRGs were dissected and post-fixed in the same fixative for 10 min. Tissue was then incubated in 20% sucrose in PBS at 4 °C overnight, embedded in tissue freezing medium (Leica, Wetzlar, Germany), and cryostat sections were cut at a thickness of 14 µm and stored at −80 °C. In situ hybridization (ISH) was performed using an QuantiGene ViewRNA Tissue Assay (Thermo Fisher Scientific, Frankfurt, Germany). Experiments were performed according to the instructions of the manufacturer and included extensive washing steps as described previously [6]. Probes specific for mouse Rab27a (catalog # VB1-3028872, Thermo Fisher, NCBI # NM_001301230.1) and scramble control for type 1 (catalog # VF1-17155) were incubated overnight at 40 °C (Thermobrite, Leica, Wetzlar, Germany). Sections were then consecutively incubated with PreAmplifier Mix QT, Amplifier Mix QT, an alkaline phosphatase labeled probe against the Amplifier, AP Enhancer Solution, and Fast Red Substrate. Finally, sections were mounted with Fluoromount G (Southern Biotech, Birmingham, AL, USA) or further processed for subsequent immunohistochemistry.

For in situ hybridization experiments combined with immunohistochemistry, hybridization of Rab27a was performed as described above. Sections were then blocked in 10% normal goat serum (NGS) and 3% BSA in PBST for 1 h and incubated with primary antibodies overnight using rabbit anti-NF200 (1:1000; Sigma-Aldrich # N4142) and mouse anti-peripherin (1:500; Chemicon/Merck Millipore, Darmstadt, Germany # MAB1527). After rinsing in PBS, slides were incubated with secondary antibodies conjugated with Alexa Fluor 488 or Cy5 (Life Technologies) for 2 h at room temperature. Nuclei were stained with DAPI (600 nM; Invitrogen # D1306) and slides were covered with mounting medium and a coverslip as described above. Images were taken using a Nikon Eclipse Ni-U microscope as described above. To analyze the distribution of Rab27 mRNA in DRG neurons, only Rab27-positive profiles with a clear nucleus were analyzed. The percentage of all DRG neurons (visualized by the autofluorescence of DRG neurons) positive for Rab27 and of Rab27-positive DRG neurons expressing peripherin or NF200 was calculated. In total >650 DRG neurons of 4 animals (at least 100 cells per animal) were counted.

### 2.5. Western Blot

Lumbar (L4–L5) spinal cords and DRGs were dissected, frozen in liquid nitrogen and stored at −80 °C until use. Samples were homogenized in Phosphosafe buffer (Novagen/Merck Millipore) combined with a protease inhibitor cocktail (Complete Mini; Roche Diagnostics, Mannheim Germany). Extracted proteins (30 µg per lane) were separated by SDS-polyacrylamide gel electrophoresis and blotted onto a nitrocellulose membrane. Membranes were blocked in blocking buffer (Odyssey blocking buffer, LI-COR Bioscience, Bad Homburg, Germany; diluted 1:1 in PBS) for 1 h at room temperature (RT) and then incubated with rabbit anti-Rab27a (1:500; Proteintech, Manchester, UK # 17817-1AP; immunogen consists of a human Rab27a fusion protein, which shows 96% homology to murine Rab27a (NCBI # AAG24638.1) but only 71% homology to murine Rab27b (NCBI # AF02885.1)) or mouse anti-GAPDH (1:2000; Ambion # AM4300) dissolved in blocking buffer containing 0.1% Tween-20 overnight at 4 °C. After incubation with secondary antibodies for 1 h at RT, proteins were detected using an Odyssey Infrared Imaging System (LI-COR Bioscience). Quantification of band densities was done using Image Studio Lite software (LI-COR).

### 2.6. Real-Time RT-PCR

Total RNA of lumbar DRGs (L4-L5) and the lumbar spinal cord (L3-L5) was extracted under RNase-free conditions using a RNAqueous Micro Kit (Ambion/Life Technologies, Carlsbad, CA, USA) or a RNeasy Mini Lipid Tissue Kit (Qiagen, Hilden, Germany), respectively. Genomic DNA contaminations were reduced by treating samples with DNase for 15 min. Reverse transcription was performed using a Verso Kit (Thermo Fisher Scientific, Frankfurt, Germany) according to the manufacturer’s instructions. Quantitative real-time RT-PCR (qPCR) was performed with an ABI Prism 7500 Sequence Detection System (Applied Biosystems/Life Technologies) using SYBR green (Applied Biosystems/Life Technologies) and gene specific primers for murine Rab27a (Fwd 5′- TTC AGG GAC GCT ATG GGT TT-3′ and Rev 5′- CCA CAC AGC ACT ATA TCT GGG-3′, NCBI # AB120511.1, nucleotides 265–395), Rab27b (Fwd 5’-TGG TCT CAA TGT CTG TGC CA-3’ and Rev 5’-AGC TCA CGA CAT TCA CCA GA-3’, NCBI # NM_030554.4, nucleotides 3017–3139) and GAPDH (Fwd 5’-CAA TGT GTC CGT CGT GGA TCT -3’ and Rev 5’-GTC CTC AGT GTA GCC CAA GAT G-3’, NCBI # XM_017321385.2, nucleotides 1145–1352). RT-PCR products generated by Rab27a or Rab27b primer pairs result in in specific products for Rab27a or Rab27b, respectively. Reactions were performed in duplicate or triplicate by incubating for 10 min at 95 °C, followed by 40 cycles of 15 s at 95 °C and 1 min at 60 °C, including water controls to ensure specificity. Relative gene expression levels were calculated using the comparative 2-ΔΔCt method and normalized to GAPDH. 

### 2.7. Statistics

Statistical analysis was performed with SPSS software using the Student’s t test for paired comparisons, or one-way ANOVA for multiple comparisons followed by a Fisher post hoc test. When mice were tested at different time points we used a repeated-measures two-way ANOVA and differences between groups at each time point were analyzed with a Fisher’s least significant difference (LSD) post-hoc test. Rotarod fall-off latencies were analyzed with Mann-Whitney U test and are expressed as median and interquartile range. All other data are presented as the mean ± standard error of the mean (SEM). For all tests, a probability value *p* < 0.05 was considered as statistically significant. 

## 3. Results

### 3.1. Rab27a Expression in the Spinal Cord and in Dorsal Root Ganglia

**Figure 1:**
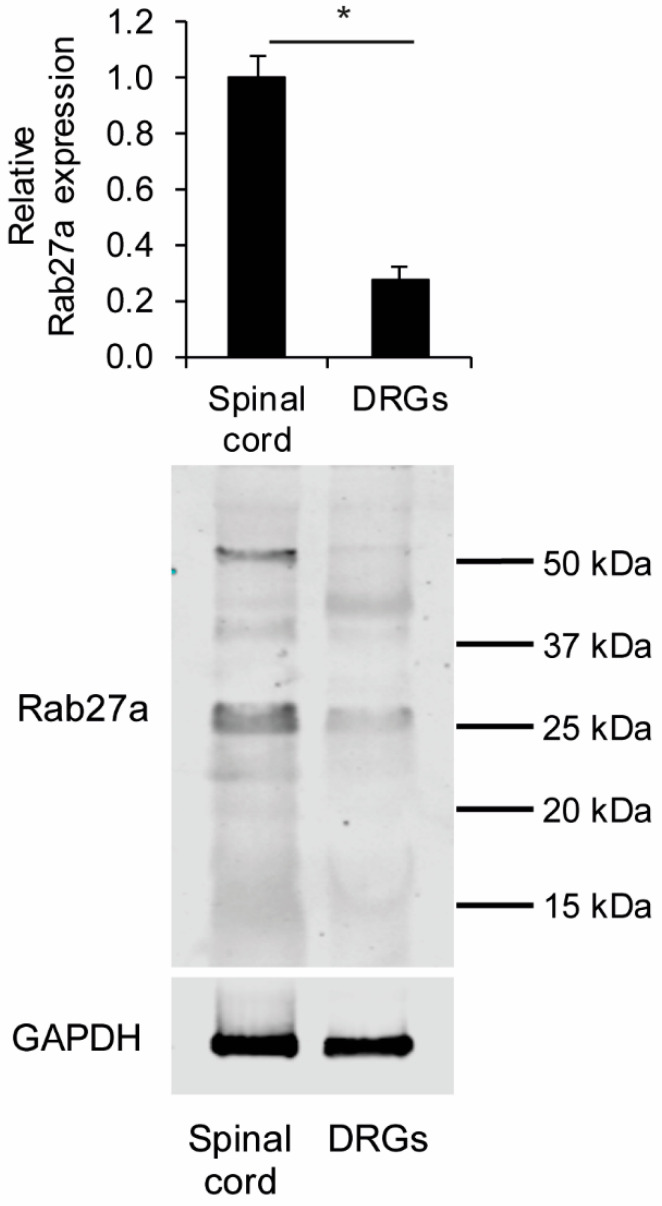
Western blot of Rab27a in the spinal cord and dorsal root ganglia (DRGs) of naive wild type (WT) mice. Rab27a expression at the expected size of 25–27 kDa is significantly stronger in the spinal cord compared to DRGs (*n* = 4), * *p* < 0.001. Some additional bands around 40–50 kDa were also detected, which seem to be unspecific.

We first investigated the expression of Rab27a in the spinal cord and DRGs of naive wildtype (WT) mice using an antibody whose specificity was previously demonstrated [41]. In Western blot experiments we detected Rab27a in both the spinal cord and DRGs at the expected size around 25–27 kDa with higher relative expression levels in the spinal cord compared to DRGs (Figure 1).

**Figure 2:**
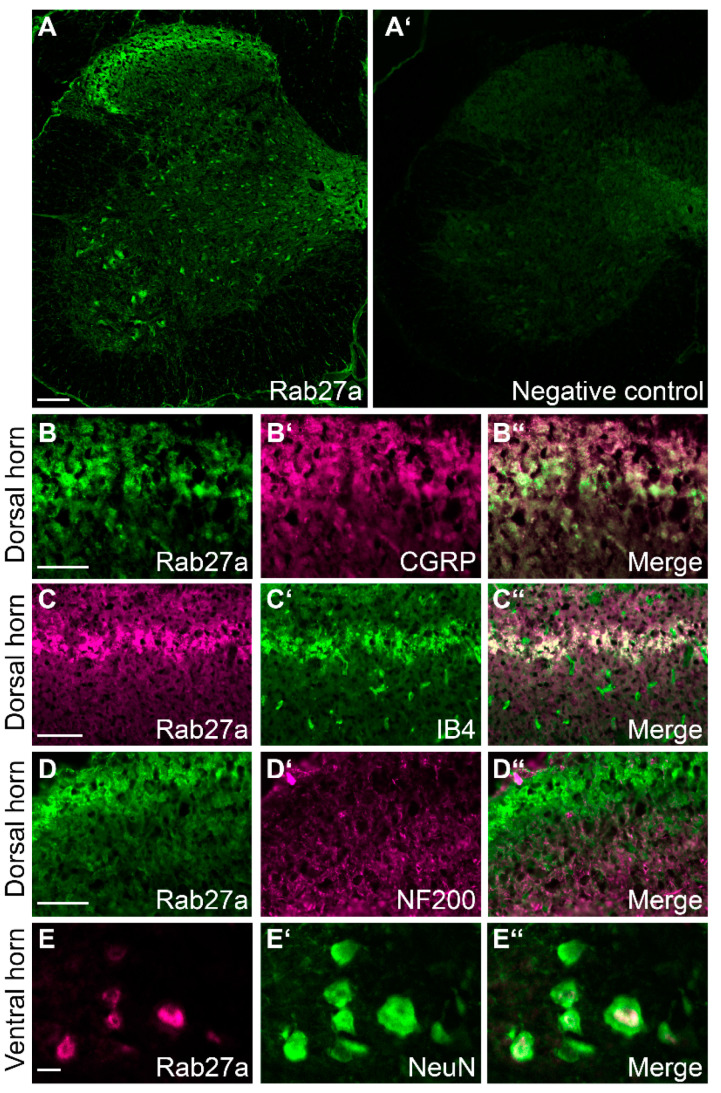
Rab27a localization in the spinal cord. (**A**) Immunostaining experiments detected Rab27a in the dorsal horn and in some ventral horn neurons. No specific signal was detected by omitting the primary antibody (**A**’, negative control). (**B**–**D**’’) Double immunolabeling experiments with established markers revealed that Rab27a colocalizes with calcitonin gene-related peptide (CGRP) (**B**–**B**’’) and isolectin B4 (IB4) (**C**–**C**’’), while there was virtually no co-expression with neurofilament 200 (NF200) (**D–D**’’). (**E–E**’’) Rab27a protein also co-localizes with NeuN, a neuronal marker, in the ventral horn of the spinal cord. Scale bars A: 100 µm, (**B**–**D**): 50 µm, E: 10 µm.

Interestingly, immunostaining experiments in the spinal cord of naive mice revealed Rab27a immunoreactivity to be enriched in the superficial dorsal horn (Figure 2A), i.e., in an area that is important for nociceptive signaling. Rab27a immunoreactivity was also detected to a lesser extent in a scattered pattern in the ventral horn (Figure 2A). As expected, no immunofluorescence was detected in control experiments omitting the primary anti-Rab27a antibody (Figure 2A’). We then analyzed the cellular distribution of Rab27a in the spinal cord using double-labeling immunostaining experiments. We observed that Rab27a partly co-localizes with CGRP and isolectin B4 (IB4), which are established markers for nociceptive peptidergic and non-peptidergic C fibers, respectively, terminating in the superficial dorsal horn (Figure 2B,C). By contrast, Rab27a is not colocalized with NF200, a marker for myelinated primary afferent fibers (Figure 2D). In the ventral horn, Rab27a immunoreactivity was found in cells expressing NeuN, a marker of neuronal somata (Figure 2E). Together, the enriched immunoreactivity of Rab27a in the superficial dorsal horn points to a contribution of Rab27a to pain processing.

**Figure 3:**
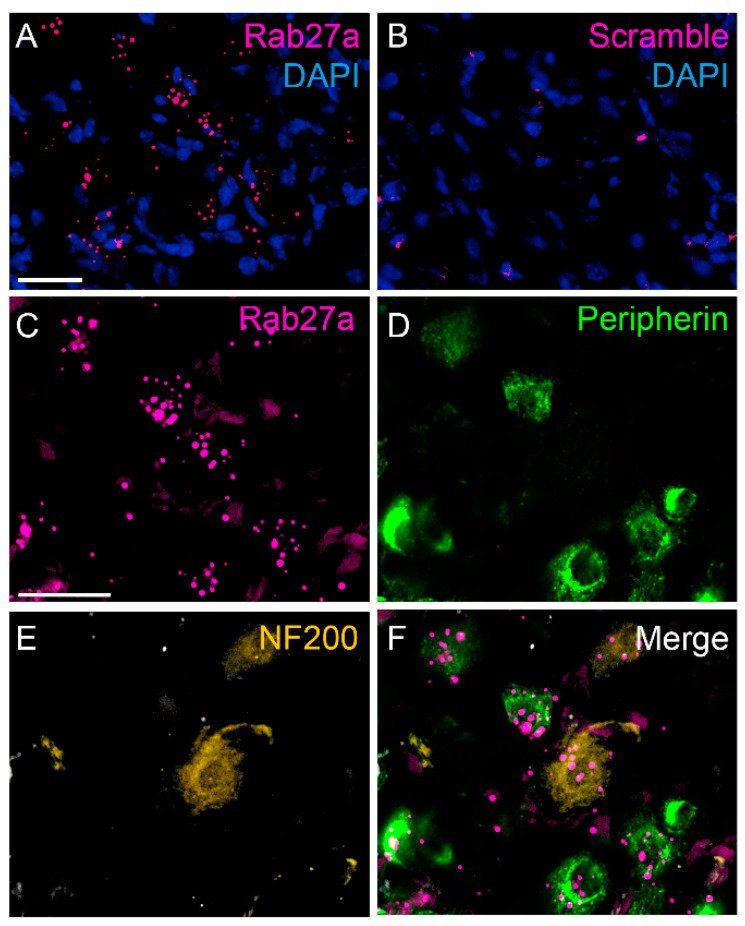
Localization of Rab27a mRNA in dorsal root ganglia. (**A**) Fluorescent in situ hybridization detected Rab27a mRNA in DRG neurons and non-neuronal cells. (**B**) No hybridization signal was detected using scramble (negative) control. (**C**–**F**) In DRG neurons Rab27a mRNA colocalizes with peripherin-positive and NF200-positive DRG neurons. Scale bars: A and C = 25 µm.

In DRGs we were not able to detect a specific Rab27a immunofluorescence signal using various staining protocols. We therefore investigated the distribution of Rab27a mRNA using fluorescent in situ hybridization (ISH). Hybridization signals for Rab27a mRNA were detected in many DRG cells in close proximity of DAPI-positive nuclei. Of note, the signal appeared to be localized in distinct compartments within the cells (Figure 3A). No hybridization signals were detected using a scramble control probe (Figure 3B). ISH combined with immunostaining revealed that 63.03% ± 10.68% of cells positive for peripherin, a marker for C-fiber neurons, also expressed Rab27a (Figure 3C–F). Moreover, 36.97% ± 10.68% of cells expressing NF200, a marker for myelinated primary afferent neurons, were positive for Rab27a. In total Rab27a mRNA was expressed in 70.70% ± 2.27% of sensory neurons. As some Rab27a hybridization signals were found in close proximity of DAPI-positive nuclei which did not co-localize with the neuronal markers, our staining further suggests that Rab27a is also expressed in non-neuronal DRG cells. Altogether, these data show an enriched expression of Rab27a in nociceptive C fiber neurons, suggesting a possible contribution to pain processing. 

### 3.2. Rab27a^ash/ash^ Mice Display Normal Basal Sensitivity

**Figure 4:**
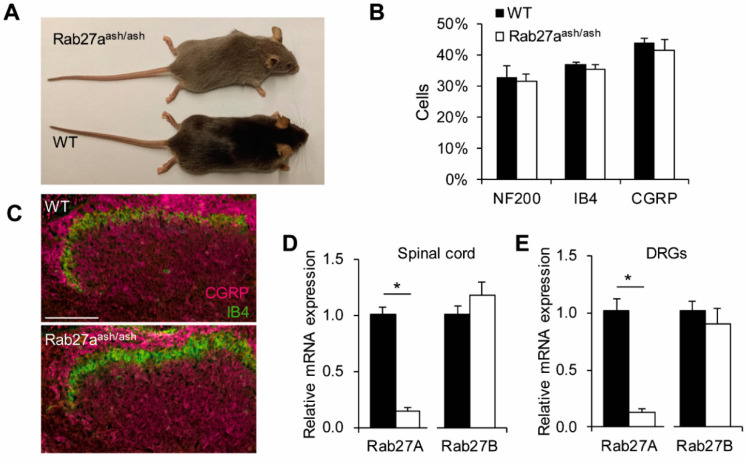
Basal characterization of Rab27a^ash/ash^ mice. (**A**) Rab27a^ash/ash^ mice exhibit a gray coat color. (**B**) Quantification of DRG neuron populations in Rab27a^ash/ash^ and WT mice. The percentage of NF200, isolectin-B4 (IB4) and CGRP-positive DRG neurons is similar between genotypes (*n* = 3). (**C**) Distribution of primary afferent terminals as assessed by the markers CGRP and isolectin-B4 (IB4) is comparable between genotypes; Scale bar: 100 µm. (**D–E**) The isoform Rab27b is not compensatory upregulated in Rab27a mutant mice in the spinal cord (**D**) or in DRGs (**E**); * *p* < 0.05.

To investigate a possible function of Rab27a in pain processing, we characterized the behavior of Rab27a^ash/ash^ and littermate WT mice in animal models of pain. Rab27a^ash/ash^ mice are viable, fertile und do not show any gross behavioral impairments. However, due to defects in pigment granule transport they exhibit a gray coat color (Figure 4A). In order to obtain reliable results, special attention was paid to blinding in the behavior experiments (see material and methods).

For basal characterization of nociceptive pathways, we analyzed the percentage of DRG neuron populations positive for the marker NF200, IB4, and CGRP and observed no differences between WT and Rab27a^ash/ash^ mice (Figure 4B). Further, the distribution of IB4-binding and CGRP-positive terminals in the superficial dorsal horn is similar between genotypes (Figure 4C), suggesting that there are no gross developmental defects affecting DRG neuron subpopulations and spinal cord morphology in Rab27a^ash/ash^ mice. The expression of Rab27b, the second Rab27 isoform, is not compensatory regulated in DRGs and the spinal cord of Rab27a^ash/ash^ mice (Figure 4D,E). Moreover, as a prerequisite for interpretation of behavioral pain studies, the motor function was not impaired in Rab27a^ash/ash^ mice as assessed by the rotarod test (mice of both genotypes reached a median fall off latency of 120 s). 

**Figure 5:**
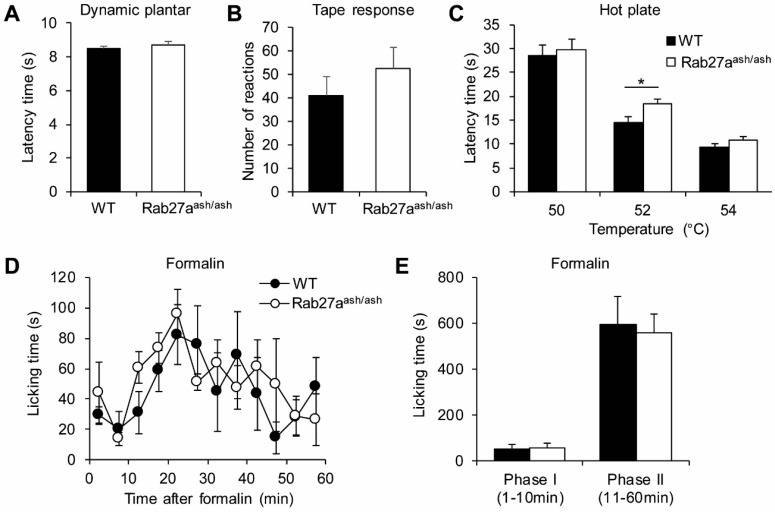
Rab27a^ash/ash^ mice show normal responses in basal mechanical and nociceptive pain models. (**A**–**B**) Reaction to basal mechanical stimuli, as assessed by the Dynamic plantar test (A, *n* = 20–22) or the tape response assay (B, *n* = 19) are not affected in Rab27a^ash/ash^ mice. (**C**) Rab27a^ash/ash^ mice show slightly reduced pain behavior in the hot plate test at 52 °C, while their responses at 50 °C and 54 °C were comparable to those of WT mice (C, *n* = 19). (**D**–**E**) Formalin-induced nociceptive pain behavior is normal in Rab27a^ash/ash^ mice (*n* = 5–6); * *p* < 0.05.

We then investigated basal sensitivity to mechanical stimuli in Rab27a^ash/ash^ mice using the Dynamic plantar test (Figure 5A) and the tape response test (Figure 5B) and found that the responses were not affected by the Rab27a mutation. Acute responses to noxious thermal stimuli were investigated using the hot plate test (50, 52, and 54 °C; Figure 5C). When the plate was set at a temperature of 52 °C, Rab27a^ash/ash^ mice showed slightly, but significantly, prolonged latencies as compared to WT mice. By contrast, at a temperature of 50 and 54 °C, the nocifensive responses were comparable between genotypes. Although there was only a weak effect, these data imply that Rab27a is involved in the immediate responses to thermal stimuli.

In order to investigate if Rab27a is involved in rapid sensitization of pain pathways, we performed the formalin test. Injection of 0.5% formalin into a hind paw resulted in the typical biphasic nociceptive response consisting of licking the injected paw. However, the licking behavior of Rab27a^ash/ash^ and WT mice was similar in both phases (Figure 5D,E). Altogether, these data suggest that Rab27a plays only a minor role in acute nociceptive processing.

### 3.3. Rab27a^ash/ash^ Mice Display Reduced Inflammatory Pain Behavior

**Figure 6:**
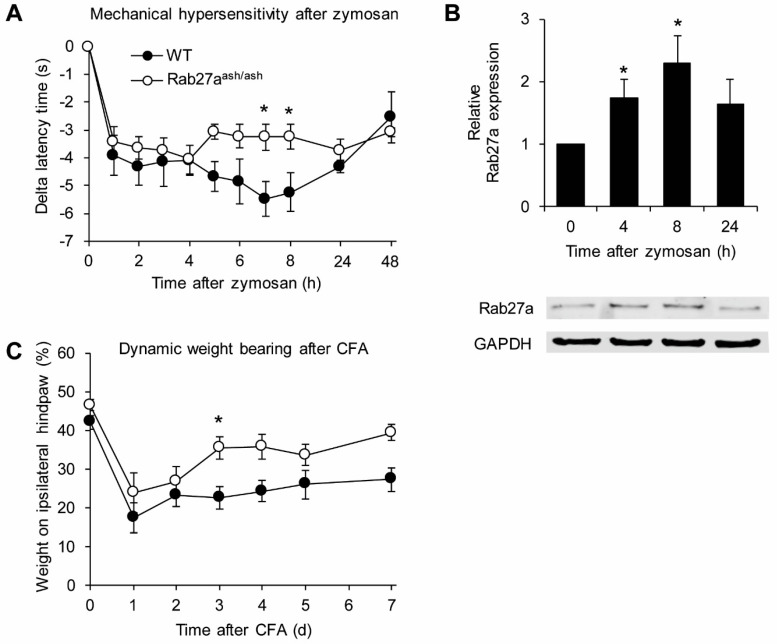
Rab27a^ash/ash^ mice show reduced pain behavior in inflammatory pain models. (**A**) Mechanical hypersensitivity after zymosan injection into a hind paw is significantly reduced in Rab27a^ash/ash^ mice compared to WT littermates (*n* = 5). (**B**) Western blot analyses revealed that Rab27a protein expression in the spinal is significantly upregulated 4 and 8 h after zymosan injection into a hind paw of WT mice (*n* = 6). (**C**) Rab27a^ash/ash^ mice show reduced pain behavior in the Complete Freund′s Adjuvant (CFA)-model of inflammatory pain as assessed by the dynamic weight bearing (*n* = 6); * *p* < 0.05.

We next investigated the behavior of Rab27a^ash/ash^ mice in models of persistent inflammatory pain. In the model of zymosan-induced paw inflammation, the injection of a zymosan A suspension into a hind paw induced a local inflammatory reaction accompanied by mechanical sensitization of the paw (Figure 6A). The mechanical hypersensitivity developed similarly over the first 4 h in both genotypes. Interestingly, however, at later stages the mechanical hypersensitivity was significantly reduced in Rab27a^ash/ash^ mice as compared to WT littermates (Figure 6A), suggesting that Rab27a is involved in pain processing in response to inflammatory stimuli. We also performed western blot analyses to investigate expression of Rab27a in the spinal cord of WT mice 4, 8 and 24 h after zymosan injection into a hind paw. Interestingly, Rab27a expression was significantly upregulated 4 and 8 h after zymosan injection (Figure 6B), further supporting the hypothesis that Rab27a plays a role in inflammatory pain. 

We then assessed the behavior of Rab27a^ash/ash^ mice in a second model of inflammatory pain. For that purpose, paw inflammation was induced by injection of CFA into a hind paw. In this model we quantified the extent of inflammatory pain using the dynamic weight bearing test, which enables assessment of spontaneous pain in freely moving animals and may thus allow for a clinically more relevant readout of pain as compared to reflexive pain tests [38]. As shown in Figure 6C, the CFA injection resulted in decreased weight bearing of the ipsilateral paw in both genotypes. However, at later time points Rab27a^ash/ash^ mice showed significantly reduced spontaneous pain behavior compared to their WT littermates. Together, these data imply that Rab27a is involved the processing of inflammatory pain.

## 4. Discussion

We here describe a previously unknown function of the small GTPase Rab27a in pain processing. Rab27a is highly expressed in nociceptive C fiber neurons and their terminals in the superficial dorsal horn of the spinal cord. Rab27a mutant mice showed normal responses to acute nociceptive stimuli, but their behavior in inflammatory pain models was significantly reduced. These data suggest that Rab27a plays a pronociceptive role in inflammatory pain models. 

The enriched expression of Rab27a in sensory neurons is supported by a recent single cell RNA sequencing study [42]. As we demonstrate that (i) Rab27a protein is expressed to a much higher extend in the spinal cord as compared to DRG neurons, (ii) Rab27a can only be detected on mRNA but not on protein level in DRGs and (iii) Rab27a protein signal is enriched in the superficial dorsal horn of the spinal cord, co-localizing with marker for primary afferent neurons, our data suggest that Rab27a protein in sensory neurons might be transported to central terminals within the spinal cord. As Rab27a is an important regulator of exocytosis processes in various cell types [43,44,45,46,47,48], it seems likely that also in the spinal cord Rab27a regulates vesicle transport or vesicle fusion in synaptic terminals of primary afferent neurons.

We show that Rab27a mutant mice displayed reduced responses in inflammatory pain models induced by paw injection of zymosan or CFA, as well as slightly reduced responses in the hot plate test. The involvement of Rab27a in inflammatory pain processing is further supported by its upregulated expression in the spinal cord after intraplantar zymosan injection, because chronic pain is accompanied by altered expression of many genes which are required for the biochemical and structural reorganization of nociceptive circuits [49]. It should be noted, however, that mutations in the gene encoding for Rab27a are possibly associated with immune defects (for review see [45]). Rab27a shows strong expression in melanocytes and lymphocytes, in which it regulates exocytosis of vesicles [13]. Thus, it plays an important role in various steps in the immune defense system such as exocytosis of azurophilic granules, gelatinase, and specific granules in neutrophils as well as in the activation of NADPH oxidase 2 in phagocytes [43]. In line with that, defects of Rab27a cause Griscelli Syndrome type 2 (GS2), an autosomal recessive pigmentation disorder that involves severe immunological defects [13]. In our study we found Rab27a to be expressed in neuronal cells pointing to a functional role in pain processing. However, as our data also suggest, a non-neuronal expression in DRGs and as Rab27a expression is well described in immune competent cells, we cannot exclude the possibility that impaired immune responses contribute to the reduced inflammatory pain in Rab27a mutant mice. However, as nociceptive behavior in the hot plate test at 52 °C was reduced in Rab27a mutant mice, this points to a potential contribution of neuronally expressed Rab27a to pain processing. 

About the mechanism by which Rab27a contributes to thermal pain and about the reason why the hot plate behavior of Rab27a^ash/ash^ mice was altered at 52 °C, but not at 50 or 54 °C, we can only speculate. In general, noxious heat sensing depends on a variety of transient receptor potential (TRP) ion channels which are expressed in different subpopulations of sensory neurons and alter their open probability in distinct temperature ranges. One well-characterized TRP channel implicated in thermal pain is TRPV1, which however only partially contributes to the nocifensive behavior in the hot plate test [50,51]. A recent study provided evidence that the combined activation of TRPV1, TRPM3 and TRPA1 plays a major role in noxious heat detection in mice [52]. However, several other ion channels have been described as molecular sensors for noxious heat including the TRP channels TRPV2, TRPV3, TRPV4, and TRPM3, as well as channels of the anoctamin (ANO)/transmembrane protein 16 (TMEM16) family [53,54,55,56,57,58], and these channels seem to contribute to the pain behavior at distinct temperatures. Hence, the altered 52 °C hot plate behavior of Rab27a^ash/ash^ mice points to a modulation of the activity of one or more of these channels by Rab27a. Of note, recent studies reported that Rab27a may modulate the activity of various channels such as the epithelial sodium channel [59], the cystic fibrosis transmembrane conductance regulator [60] or Ca_V_1.3 channels in the retinal pigment epithelium [61]. Thus, it seems possible that Rab27a interacts with heat-sensing channels in sensory neurons. 

Despite its expression in various cells, mutations of Rab27a seem to affect mainly functions of melanocytes and cytotoxic T-lymphocytes. One explanation might be a compensatory effect of Rab27b, the second Rab27 isoform which is found in vertebrates. Rab27a and Rab27b show 71% identity in their amino acid sequence and share similar effector domains and carboxy-terminal isoprenylation motifs; however, they share little sequence identity after the GTP-binding domain IV [62]. Interestingly, Rab27a and Rab27b are co-expressed in various secretory cells except melanocytes and cytotoxic-T-lymphocytes [26,63]. Thus, they were originally thought to function in a redundant manner. However, later studies revealed that the functions of Rab27a and Rab27b differ from each other as these isoforms interact with different effectors [26,44,64,65,66,67]. In this study we found the isoform Rab27b also to be expressed in the spinal cord and in DRGs, in line with previous studies [42]. However, we detected no compensatory regulation of Rab27b in Rab27a^ash/ash^ mice. Despite the fact that Rab27a mutant mice show reduced heat pain and inflammatory pain behavior, we cannot exclude the possibility that Rab27b at least partly rescues Rab27a functions in Rab27a^ash/ash^ mice. 

Altogether, our data suggest that Rab27a is involved in nociceptive processing. The insights reported here can help us to better understand pain and stimulate further studies to elucidate the functions of Rab proteins in this context.
